# Innovations in Calculating Precise Nutrient Intake of Hospitalized Patients

**DOI:** 10.3390/nu8070412

**Published:** 2016-07-04

**Authors:** Sheila Cox Sullivan, Melinda M. Bopp, Dennis L. Weaver, Dennis H. Sullivan

**Affiliations:** 1 VISN 16/CAVHS Geriatric Research Education and Clinical Center (GRECC); 2200 Fort Roots Drive, 3J/GRECC; North Little Rock, AR 72114, USA; melinda.bopp@va.gov (M.M.B.); sullivandennish@uams.edu (D.H.S.); 2 INFO Development Systems, University of Arkansas for Medical Sciences, Little Rock, AR 72205, USA; weaverdennisl@uams.edu; 3 Donald W. Reynolds Department of Geriatrics, University of Arkansas for Medical Sciences, Little Rock, AR 72205, USA

**Keywords:** nutrition assessment, nutrition status, nutritional deficiency, undernutrition, diet

## Abstract

Obtaining a detailed assessment of a hospitalized patient’s nutrient intake is often critically important to ensuring the patient’s successful recovery. However, this process is often laborious and prone to error. Inaccurate nutrient intake assessments result in the inability of the healthcare team to recognize patients with developing nutritional deficits that contribute to delayed recovery and prolonged lengths of stay. This paper describes an innovative, easy to use system designed to increase the precision of calorie count reports by using a combination of photography, direct observation, and a specially developed computer program. Although the system was designed specifically for use in a Department of Veterans Affairs Hospital, it has the potential to be adapted for use in other hospital environments.

## 1. Introduction

An essential component of therapeutic nutrition monitoring is a precise assessment of each patient’s nutrient intake [1]. However, the process of obtaining such information is very laborious. Consequently, many institutions rely instead on crude estimates obtained by the clinical staff of what percentage of each delivered meal a patient consumed. In addition, nursing staff commonly delegate the responsibility of performing such “calorie counts” to the least educated member of the healthcare team who may not comprehend the critical nature of this assignment [2,3]. Studies indicate that the resulting estimates of nutrient intake are often inaccurate, most commonly overestimated by greater than 15% [2,4,5]. Due to the lack of detailed nutritional assessments, the healthcare team may not identify patients who do not consume an adequate amount of nutrients to meet even their basal requirements; this leaves these patients at high risk for the development of potentially serious nutritional problems while hospitalized [6,7,8,9,10]. To alleviate this problem, innovative methods are needed that make it easier for the clinical staff to obtain precise estimates of a patient’s daily nutrient consumption. 

One method to improve the accuracy of calorie counts is the use of photography to verify what food the patient receives and how much of that food the patient eats [2,11,12,13,14,15,16,17,18,19,20]. This method involves taking photographs of a patient’s tray before and after the meal. By comparing these before and after photographs, a trained staff member can calculate the patient’s nutrient consumption with much greater accuracy than by using routine methods of obtaining such estimates [2,12,15]. Previous studies indicate estimates based on photographs correlate well with calorie counts obtained by highly trained specialized teams, although not with estimations by nursing staff using standard methods [2,12,15]. Photography also compares well with weighing the provided food before and after the meal [2,11,12,14,15,16,18]. However, the photographic method has a number of significant limitations. One is the inability to detect the addition or subtraction of foods from the patient’s tray between photos. A second limitation of photographs is failure to depict the composition or amount of every food item provided or the percentage of each food item consumed, particularly for those items in opaque containers. Most significantly, in order to calculate total macro- (i.e., protein, energy, and fat), and micronutrient (e.g., vitamin and mineral) composition of the consumed food, staff must undertake an arduous process including estimation of all the aforementioned parameters, identifying the nutrient content of each food item using a large database, entering the resulting values in a spreadsheet, and adding all of the individual estimates. This process takes significant time and human resources, which considerably deters the collection of precise calorie counts.

To address these issues, we developed a multi-component system for obtaining calorie counts rapidly and reliably within a Department of Veterans Affairs hospital. This system was developed to support several large ongoing clinical research studies. However, it could be adapted easily to routine clinical practice within the same hospital. With modification of the computer program, the system has the potential to be disseminated to other facilities. This article provides a detailed description of the system.

## 2. Materials and Methods 

### 2.1. Overview

The multi-component system combines a computer program, direct observation, and photography. The computer program generates a detailed listing of all food items scheduled to be served to a given patient at a given meal as per the listing on the food services menu. After the meal, the program automatically calculates macro- and micronutrient consumption based on staff-provided estimates of percentages of each food item a given patient consumed. 

### 2.2. The Computer Program

The computer program, which we developed in Microsoft Access, reduces the work required to obtain highly detailed calorie counts. The user interface provides the ability to: (1) generate menu-specific Nutritional Intake Assessment Forms (NIAF) for selected patients based on their prescribed diet, the menu cycle, and the day of the given menu cycle; (2) edit any given NIAF; (3) calculate and report detailed calorie counts; (4) add new food items or edit the nutrient composition of any food items already in the system; and, (5) edit/update existing dietary menu templates from which the NIAFs are generated. The program has multiple layers of user permissions, which prevent the individuals accessing the program from inadvertently altering or exporting protected information.

To build the computer database, we obtained the dietary menus the facility followed, which consisted of a three-week menu rotation accommodating approximately twenty different types of diets. Some of these diets may be further modified (e.g., varying levels of calorie or sodium restriction). Each menu the facility provided contained a detailed list of each food item included in each meal and a code linking that food item to a large national nutritional database. All nutrient data stored in the database were originally obtained from a modified food nutrient database from the United States Department of Agriculture (USDA) Handbooks #8 and #456 [21,22]. Through this linkage, we entered the nutritional composition of each food item into the computer, which made it possible for the computer program to calculate detailed calorie counts. This database can be updated as the USDA releases new handbooks. 

**Figure 1:**
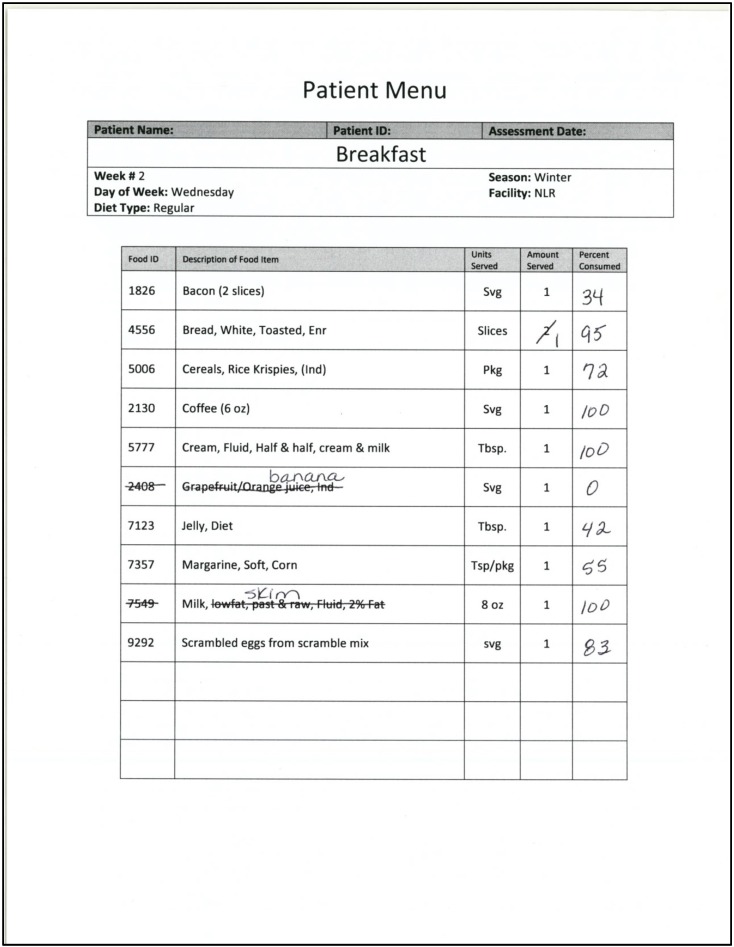
A picture of a Nutrition Intake Assessment Form (NIAF), which lists the foods scheduled to be served for a given meal based on the published hospital menu plan, showing the end-user handwritten modifications indicating what foods were actually served.

For each meal, for every prescribed diet, for each dietary cycle/rotation plan, the programmer created a template listing the specific components of the meal. This template has four columns such that the first column includes a detailed description of each food item that would be served; the second column indicates the unit of measure for the given food item; the third provides the number of units to be served (i.e., units per serving); and the fourth column provides space for the end user to write the percentage of the given food item consumed by the patient (see Figure 1). The computer program uses this template to generate the NIAF for a given patient based on the specific meal selected.

A responsible employee (RE) uses the NIAF to verify what food items were served and the percentage of each item consumed by the patient. The computer program enables the RE to preprint an NIAF for each patient for each meal of any given day. To print the NIAF, the RE selects the patient from those entered into the computer program (or registers the patient if not already in the system), the prescribed diet, and the day of the given menu cycle. Once the RE completes this input, the program generates an NIAF for each of the three meals of that day (breakfast, lunch, and dinner), automatically saves the NIAFs to a database under the specific patient’s name, and prints a copy. At this point, the NIAF is a copy of the meal template that the system programmer created for the given meal as previously described. When preparing to conduct the calorie counts, the RE takes the NIAF to the ward before the start of the meal. This provides the RE the opportunity to verify that the delivered meal is identical to what is listed on the NIAF, which is identical to the official hospital menu. As the kitchen staff may make unannounced changes to a given day’s menu, the RE corrects the entries on the given patient’s NIAF so that the entries match the actual content of the tray in terms of both the food items included and the quantity (i.e., number of servings) of each item provided. This can be done by crossing out the food items on the NIAF that are not served and entering any unlisted items directly above the crossed-out item or in one of the blank rows on the form. At this point, only a general description of any unlisted food item is necessary. One advantage of this feature is the ability to enter foods brought from outside the facility by family members, such as a breakfast item from a fast food restaurant. Once all needed corrections have been made, the NIAF is used by the RE when conducting the calorie count to record the percentage of each food item consumed by the given patient. As described below, the RE performs the calorie count following a clearly delineated process that allows for further verification of all data entered. 

Once the meal is complete and the paper copy of the NIAF is completed appropriately, the RE enters these changes to the electronic version of the NIAF previously saved under the patient’s unique identifier. Instead of using paper copies of the NIAF when conducting calorie counts, the entire computer program can be loaded on a VA approved laptop or other approved portable device allowing for a paperless process. This allows the RE to fill out and edit the electronic version of the NIAF directly.

Once the data is entered, the RE can generate the actual calorie count by individual meal or for the entire day for the given patient. The RE does this using another menu option on the computer program’s user interface. The calorie count report is created by the computer program by combining the data stored on the completed NIAF with the internal database that lists the nutrient content of all food items. This report lists the total amounts of 60 different macro- and micro-nutrients that were included in the given meal(s) and the amounts of each of these nutrients that were consumed; this includes total calories, protein, fat, carbohydrates, and many vitamins and minerals. The system allows the RE to view and/or print the calorie count report, which can be given to a healthcare provider to analyze the adequacy of the diet served and the patient’s level of consumption.

The dietary department changes the menu rotations based on summer and winter seasons so the menus templates require updating semiannually. The program allows users with appropriate permissions to make global modifications to the menu templates, which includes the ability to eliminate food items and/or enter new foods as required. In the unlikely event a food item being added to a template is not already in the program’s nutrient database, it must be added to this database first. This is completed by pulling the nutrient composition of the food item from the USDA database as described above.

Many patients receive nutritional supplements or extra food from a variety of sources. Therefore, the next process we developed tracked food or nutrition supplements provided to the patients outside the normal mealtime. The development team created a snack form to place in the patient’s bedside table. Using this form, the staff records a description of each food item (e.g., 99-g container of chocolate pudding), how many units the patient receives, and how much of it the patient eats. The patient and/or family can also be a source for this information. Each snack sheet contains space adequate to list all food items that the patient eats during a 24-h period not listed under the regular facility dietary menu for that day. A separate document captures enteral and parenteral nutrition prescribed for the patient. The parenteral worksheet includes amino acids, lipids, and dextrose, while the enteral worksheet lists the specific formulation(s) and/or amount of free water ordered. Either the staff or the RE completes these worksheets, and the RE collects the information for entry into another interface with the computer program.

As described, the computer program is a powerful tool to assist with calorie count reports of nutrient intake for patients at risk of nutritional deficits. However, the program relies on the correct assessment and data entry of food eaten to create these reports. To ensure the highest possible level of accuracy, we incorporated both direct observation and photographic methods in the calorie count data collection process. 

### 2.3. Direct Observation 

Repeated observations of a patient’s meal tray are a crucial part of the calorie count process. The healthcare team determines which patients would benefit from calorie counts and enters the appropriate orders into the electronic medical record. The nurse transcribes the order and notifies the RE that a new patient needs a calorie count. The RE prints out the appropriate NIAF for each patient on a calorie count list, and brings the NIAF to the ward prior to the anticipated delivery of meal trays. Before meals are delivered, the RE selects the trays of the monitored patients and carefully compares what is on each individual patient’s food tray to what is listed on the NIAF. As previously described, the RE edits the NIAF as necessary to make a 100% match between the foods and quantities of those foods on the tray and the NIAF. Before delivering the trays to each patient, the RE leaves a flag on the trays to notify the unit staff that a calorie count is active for this meal and this patient. Whether the patients eat in their rooms or in a common area, the RE makes periodic spot checks during mealtime and confers with the clinical staff to verify when a given patient has food items added to or removed from his/her meal. Because patients are encouraged to ask for extras of certain foods, the staff alerts the RE Team to this by leaving the label or packaging of the food item on the tray. Having the REs present also ensures they are able to finish the assessment before staff returns the trays to the kitchen. When the patient is finished eating, the RE does a final check to ensure that the NIAF correctly lists the name and number of servings provided for all of the food items delivered to the patient. Using the corrected NIAF to record the results, the RE looks at each food item on the tray and then records the percentage (amount eaten/total amount served) of that item consumed by the patient. The process we devised for RE training and the reasons why we had the REs make their estimates on a continuous numerical scale (as opposed to the nearest 25th percentile) is detailed in the accompanying article [23]. 

### 2.4. Photography

**Figure 2:**
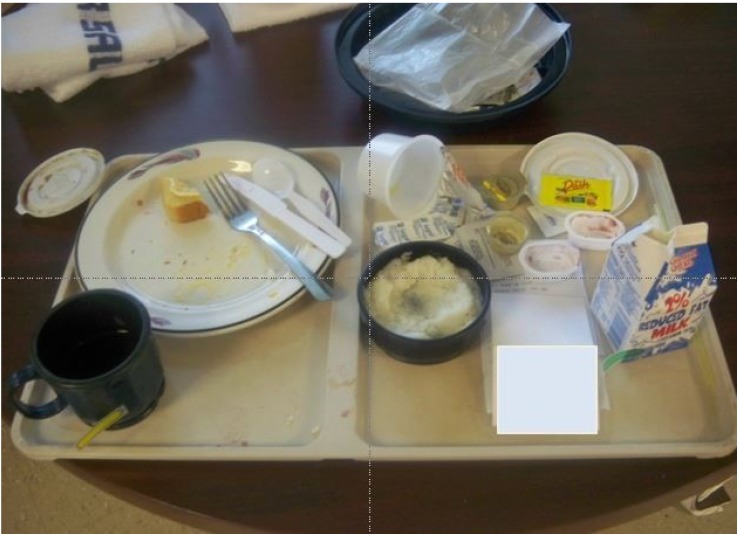
A patient’s tray after eating. Note the overturned container to communicate it is empty and the flags on the milk carton and coffee cup to show the level of the remaining fluid in the container.

The second step in ensuring precise documentation is photographing the food before and after the patient eats. Our team followed the protocol outlined by Simmons et al. [2], except we took before pictures on the unit rather than in the kitchen. While the RE removes a tray from the dietary cart and compares it to the NIAF, they ensure that all food items (including any condiments such as jelly or catsup) are included in the nutrition evaluation and are readily visible. The RE team has developed a method for communicating information via the pictures. For example, a milk carton placed on the tray horizontally signals it is empty, while a partially filled container remains upright with a sticky flag placed at the fluid level to indicate clearly in the picture the amount consumed (see Figure 2). The RE downloads images from the camera after every meal and transfers them to a PowerPoint ™ file so that each meal is in a distinct electronic file. Each patient has an electronic file under which individual meal files are stored. The naming convention for the files includes the date and meal depicted in the file. After the meal concludes, the RE enters all of the data from the corrected NIAF into the computer program. This includes their estimates of the percentage of each food item delivered to the patient that was eaten. The methods we used to ensure that these estimates are reasonably precise are described in the accompanying paper [23].

## 3. Results

Once developed, the multi-component method for obtaining nutrient intake assessments was successfully utilized in support of a large 5-year nutrition study within a United States Department of Veterans Affairs hospital. The REs successfully utilized the system each day to print copies of the appropriate NIAF for each subject on the study. Utilizing the NIAF, each RE was able to complete detailed nutrient intake assessments on up to 15 subjects at any given meal, especially if all of the subjects were on the same ward. Using small digital cameras, the REs also took before and after meal photographs of each tray. These photographs were used for quality control purposes as described previously. Once all of the data from each of the completed NIAFs was entered into the specialized computer program and the entries verified using the photographs, the REs were able to produce detailed calorie count reports that include a complete listing of the total amounts of energy, protein, fats and select micronutrients consumed by the given patient at the given meal. These reports were stored in the computer for later analysis. Each morning prior to the start of breakfast, a detailed nutrient intake assessment report covering the prior 24 h was generated for each subject that had calorie counts performed the prior day. This report was delivered immediately to the subject’s clinical healthcare team.

As per design, the computer program allowed each menu or meal plan to be edited easily thus allowing for rapid accommodation to temporary or permanent changes to the hospital’s dietary menu. Although non-institutional food items brought in from outside the hospital had to be added to the computer program, this process was also easily and rapidly completed by the REs when necessary.

Other than keeping the menus up-to-date, no other alterations were needed to maintain the functionality of the multi-component method for obtaining nutrient intake assessments. In an accompanying paper, we report the results of validation studies of the Multi-Component Method and discuss its strengths and limitations [23].

## 4. Discussion 

Digital photography has numerous advantages, and one of the most important is immediate access to the images. This instant access permits REs to evaluate the photograph’s quality and the clarity of the food items on the tray. If the image is unsatisfactory, the RE takes another photograph. An additional benefit from the digital images is the ability to use them immediately for verification of the direct observation as described above. We also use the photographs for RE training and ongoing quality assessments (by allowing supervisors to verify the work of REs).

Although this system was developed for use in one hospital facility, it could be exported to other facilities within or outside of the Veterans Health Administration. The main challenge would be to modify the computer program to include the dietary menu of the specific hospital facility. Commercial vendors may be interested in developing this capability. All other components of the system are easily exported. Very inexpensive and readily available digital cameras can be utilized for the pictures (or even a smart phone), and a variety of personnel can be utilized to implement the system. We utilized unlicensed assistive personnel whom we found could make reasonably precise estimates of nutrient intake once properly trained. Details of the RE training program, system validation, and comparisons of the time required to complete detailed nutrient intake assessments using this system compared to more traditional methods are provided elsewhere [23].

## 5. Conclusions 

This paper describes a multi-component system for obtaining calorie counts that combines a computer program, direct observation, and photography. We believe this system greatly improves the process of obtaining precise estimates of a hospitalized patient’s nutrient intake. In so doing, it meets a critical need in providing meaningful nutritional reports to providers that can guide nutritional therapy and foster optimal patient care.
